# Preparation and benchmarking of novel cellulose nanopaper

**DOI:** 10.1007/s10570-022-04563-0

**Published:** 2022-04-15

**Authors:** Wriju Kargupta, Reanna Seifert, Mark Martinez, James Olson, Joanne Tanner, Warren Batchelor

**Affiliations:** 1 Department of Chemical Engineering, Bioresource Processing Research Institute of Australia (BioPRIA), Monash University, Melbourne, VIC 3800 Australia; 2 Pulp and Paper Centre, The University of British Columbia, 321-2385 East Mall, Vancouver, BC V6T 1Z4 Canada

**Keywords:** Water vapour permeability, Oxygen permeability, Refining, Nanopaper

## Abstract

Synthetic polymers and plastics which are currently used as barrier materials in packaging applications are neither renewable nor biodegradable. Nanopaper, which is obtained by breaking down cellulose fibers into nanoscale particles, have unique properties with the potential to replace synthetic packaging materials, but requires very high energy to mechanically process the fibers into nanopaper. This research investigates whether refining alone can be used to produce nanopaper with sufficient quality for packaging applications. Nanopaper was produced from Bleached Eucalyptus Kraft (BEK) refined with a PFI mill and from Northern Bleached Softwood Kraft (NBSK) refined in a pilot disc refiner. Both trials found a plateau for oxygen permeability and water vapour permeability that was reached after 1800 kWh/t and 12,000 kWh/t for refining in the pilot disc refiner and PFI mill, respectively. Refining beyond these optima produced either little or no reduction in permeability, while increasing the drainage time to form a sheet. However, elastic modulus, strain at break and sheet light transmittance did continue to increase. The plateau oxygen permeability of ~ 1.24 (cc µm)/(m^2^ day kPa) is 1–3 orders of magnitude lower than the oxygen permeability for PET and LDPE, respectively, while the plateau water vapour permeability ~ 3 × 10^–11^ g/m.s. Pa was 1–2 orders of magnitude higher than for PET and LDPE. The improved strength and barrier properties of nanopaper achieved at lab and pilot scale mechanical refining process promises a sustainable alternative to conventional packaging.

**Graphical abstract:**

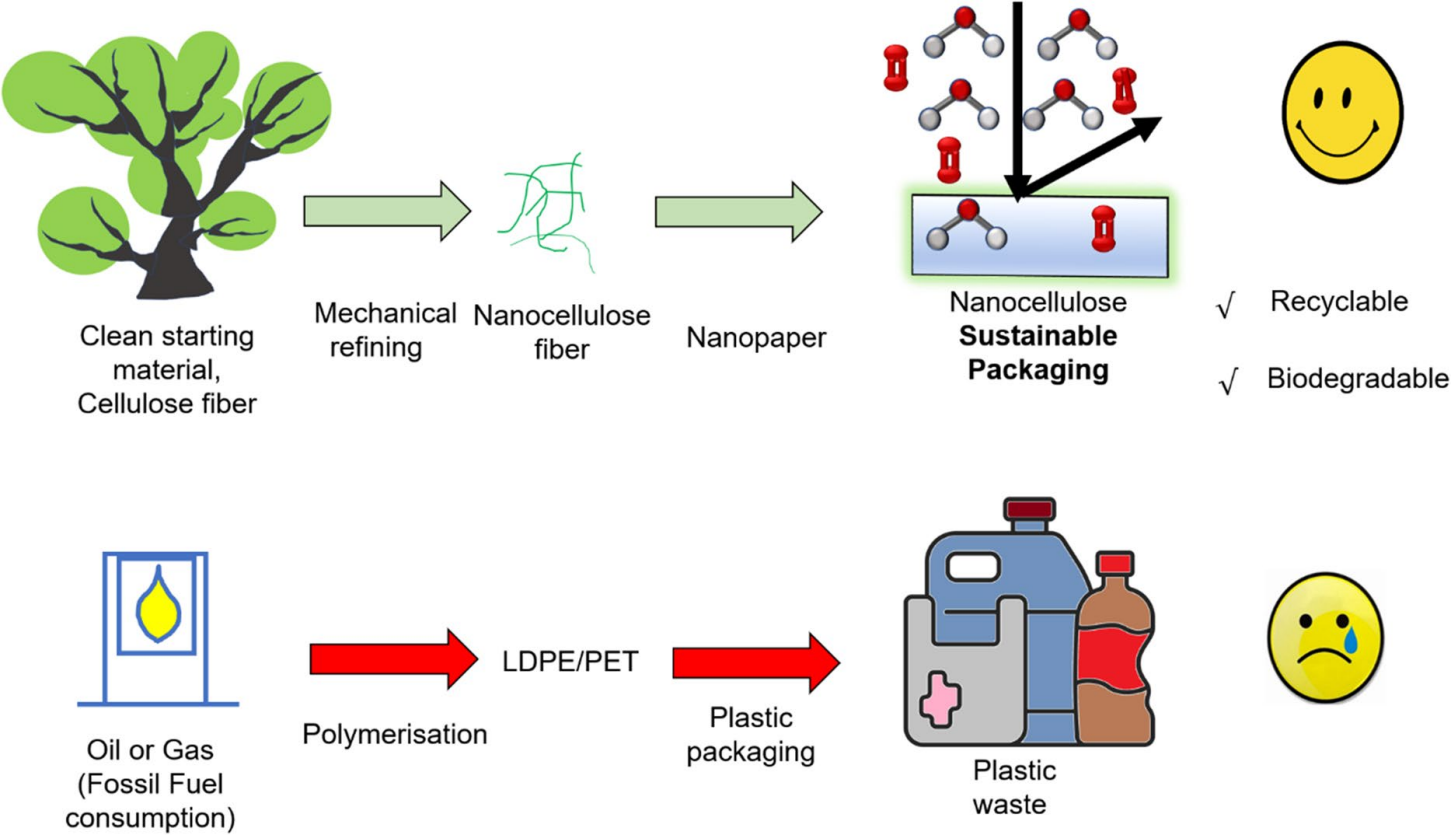

**Supplementary Information:**

The online version contains supplementary material available at 10.1007/s10570-022-04563-0.

## Introduction

The impact of COVID-19 has created many social changes escalating the plastic consumption which has inevitably led to incorrect waste disposal and inadequate waste management (Fan et al. 2021). The pandemic has increased the usage of personal protective equipment (PPE), such as gloves, face shields and masks driving the packaging industry to explore low cost, renewable materials for minimizing soil, air and water pollution (Robaina et al. 2020). Packaging plays an important role in the food supply chain; protecting the food is critical, from manufacturing, processing till end of logistics and use by consumers. The global consumer packaging market is currently in the range of US400b–$500b and is one of the fastest growing markets (Huang et al. 2019). Currently around 85%, largest share of packaging sector is occupied by food packaging (Silva et al. 2020). However, most packaging industries rely on polymers such as polyethylene (PE), polypropylene (PP), polystyrene (PS), and polyethylene terephthalate (PET) as gas barrier materials. However, these materials are petrochemical-based and non-biodegradable, and once integrated into packaging they are also typically non-recyclable. Nanocellulose derived from wood materials is a potentially market-changing feedstock for packaging materials that stands out as highly recyclable, sustainable, with excellent moisture and air barrier performance for applications in the food packaging industry (Ang et al. 2021; Ferrer et al. 2017). Nanocellulose can also be a candidate for replacing plastics for humidity, grease and oxygen barriers (Hubbe et al. 2017). Thus, nanocellulose has been a target of extensive research for food packaging as it poses very minimal threat to the environment and is manufactured from sustainable and renewable resources (Han et al. 2018).

Nanocellulose, has seen increased attraction towards packaging (Hubbe et al. 2017). Cellulose is renewable, biodegradable and abundant. It can be derived from virtually any natural biomass. The stable and stiff chemical structure of cellulose is due to the linear chain of glucose units packed by β 1, 4 glycosidic bonds aggregated together via hydrogen bonding and Vander Waals forces (Miao et al. 2020). Despite of inherent hydrophilicity of cellulose, it has unparallel advantage for usefulness in packaging and coating industry owing to its abundance, surface hydrophobicity, biodegradability and lightness in weight compared with non-renewable polymers (Wei et al. 2020). When cellulose fiber is refined, or homogenised at high pressure, low diameter, high aspect ratio nanocellulose fibers are produced. This threefold reduction in scale of fiber diameter from micron to nanometer scale comes at a cost of high energy consumption when nanocellulose are prepared in lab scale (Ang et al. 2019a).

The fiber diameter reduction from cellulose (µm) to nanocellulose (nm) promotes the production of a unique class of material (nanocellulose) having high specific surface area, high elastic modulus, high thermal conductivity, light weight, good biodegradability, and excellent barrier properties (Jiang et al. 2021; Osong et al. 2016; Thomas et al. 2020). Nanocellulose has large specific surface area and also the ability to form inter and intra molecular hydrogen bond which allows the nanomaterial to form a dense and strong network making it very hard for various molecules (oxygen and water) to pass through (Wu et al. 2022).

Nanocellulose thus produced can be processed into films and membranes with high strength and advantageous thermal, moisture and gas barrier properties (Lavoine et al. 2012). Nanocellulose thus represents a potential alternate material to the current synthetic polymers employed as barrier materials in packaging applications such as food packaging. There have been extensive studies conducted on the use of nanomaterials in nanopaper production. Nanopaper is any cellulose-based non-woven material comprising partly or wholly of nanoparticles. These nanomaterials include both nanocellulose and other nano-scale particles, all of which add desirable properties to the nanopaper such as high strength and low weight, making nanopaper useful in packaging applications (Barhoum et al. 2017). The use of cellulose nanopaper has been most widely studied in terms of mechanical performance and as nanocomposites (mixing nanopaper with counterions, polymers and nanoclays) (Benítez & Walther 2017). Nanopaper has been proposed to be used as food packaging, barrier coating or as a reinforcement with other biopolymers (Trifol & Moriana 2022). Nanopaper is transparent, shows excellent mechanical properties and barrier properties which are so critical and fundamental in food packaging applications as moisture, oxygen of the environment can deteriorate the durability and shelf life of the food (Azeredo et al. 2017). The durability and prolonged shelf life of nanopaper protect products in the circulation process, facilitate storage and transportation, and promote sales promising a renewable alternative to synthetic polymer packaging materials. The other advantage of using nanopaper that fits the green packaging sustainability design are light weight, renewability, biodegradability, and recylability (Shanmugam et al. 2019).

Recently, the effect of posttreatment (mechanical grinding with the enzyme endonuclease) on nanocellulose paper properties was also investigated (Xu et al. 2021). However, whether the energy and cost of production of nanocellulose matches with that of polymers remains to be explored. The effect of energy input to the final properties of nanopaper and its implication on the nanopaper packaging properties has been occasionally studied (Bharimalla et al. 2017).

The use of nanocellulose as a reinforcing agent and in biocomposites to improve mechanical strength and barrier properties has been also investigated for packaging applications (Youssef et al. 2012). The Young’s modulus of an alginate nanocomposite increased from 150 to 300 MPa with increasing nanocellulose content from 0 to 5 wt% (Abdollahi et al. 2013). The mechanical properties of starch-gelatin-nanocellulose films were also studied with increases in gelatin and nanocellulose concentration increasing tensile strength, which indicates better film resistance and is applicable to the packaging industry (Alves et al. 2015). Bio-nanocomposite films of carboxymethyl cellulose (CMC) and starch reinforced with nanocellulose also showed increased tensile strength with 5 wt% addition of nanocellulose from 66.82 to 110 MPa (El Miri et al. 2015). As well as imparting mechanical strength, nanocellulose addition to composites provides a physical barrier, creating a tortuous and longer diffusion pathway for moisture or gas molecules permeating across the membrane, thereby improving barrier properties (Paralikar et al. 2008). The addition of 3 wt%, 6 wt% and 9 wt% nanocellulose as a filler by improved decreasing water vapor permeability by 7%, 20% and 29% respectively (Paralikar et al. 2008). Packaging of dried fruits for instance nuts, spices, requires less moisture content barrier have been obtained through multilayer packaging. Nanocellulose when layered on polyethylene terephthalate along with low density polyethylene (LDPE) giving an improved barrier performance than commercial ethylene vinyl alcohol multilayered film (Vartiainen et al. 2014). Nanocellulose also has been also used as coatings on paper to increase the barrier properties and moisture resistance. Along with the biodegradability and environmental benefits of using nanopaper, the packaging and biodegradability properties of nanocomposites (polyvinyl alcohol filled with nanocellulose) have also been explored recently to highlight their beneficial properties in terms of enhancing the quality of packaged materials and prolonging their shelf life (Azmin et al. 2020; Oyeoka et al. 2021).

Most studies to date have assessed the merits of using commercially available nanocellulose as a barrier enhancing material in biocomposites or as a reinforcing agent (Luo et al. 2019; Mondragon et al. 2015). There has been no study reported which has investigated the correlation between nanocellulose refining type or level with pulp type and barrier properties. Refining of cellulose pulp into nanocellulose is a potentially important process for the production of nanocellulose for packaging applications, as it produces an entangled network of nanofibers which imparts improved barrier properties (Nair et al. 2014).

The properties of pure nanocellulose films have been investigated, with water vapor permeability reported being 8.12 × 10^–11^ g/m.s Pa in comparison to 8.75 × 10^–13^ g/m.s Pa for a low-density polyethylene film (Rodionova et al., 2011). Thermal treatment has also been explored which decreased water vapor and oxygen permeability by 50% and 25% respectively while treating nanocellulose film at 175 °C for 3 h (Sharma et al. 2014). There have also been attempts to improve the barrier performance of nanocellulose films by chemical modification. Nanocellulose films have excellent oxygen permeability around 0.6 cc µm/m^2^ day kPa which is an order of magnitude lower than petroleum based barrier materials such as PET (10–50 cc µm/m^2^ day kPa) (Lange & Wyser 2003; Österberg et al. 2013). While these results indicate a promising outlook for nanocellulose as packaging material; uncertainty still exists in how pure nanocellulose film develops for a given amount of mechanical treatment (Rodionova et al. 2011). There has been no study reported which has investigated the effect of refining type and refining level on nanocellulose property development on barrier properties and potential packaging applications.

Typically, pre-treatment and posttreatment are done to improve the quality of final nanopaper however the significance and novelty of our research is that mechanical refining is used as a standalone in lab and pilot scale without additional stages. Refining of cellulose pulp into nanocellulose is a potentially important process to produce nanocellulose for packaging applications, as it produces a series of small fibers and fines. Mechanical treatments such as PFI mill or disc refining work on the principle of subjecting fibers to large shear forces which fibrillate the microfibrils, enhancing the fiber bonding, and leads to the formation of dense structure. The delamination and peeling of the cell wall structure of cellulose increases the specific surface area and relative bonded area (Stankovská et al. 2019). This could potentially impart improved diffusion pathways through the network, enhancing moisture and gas barrier properties. Very few studies have been conducted which have correlated the beating degree in PFI mill in terms of refining energy consumption and compared that with the final properties of nanopaper. Unlike typical lab scale disintegration, PFI mill refiners beats pulp at 10 wt% and creates high shear promoting peeling of cell wall, fines generation, improving the fiber bonding ability, sheet density and aspect ratio. Nanofibrillation of pulp fibers have also been explored with the help of grinders (Lavoine et al. 2012). However, these stone grinders suffer from the limitation of huge energy consumption and generation of abrasive shear forces which leads to fiber fracture and breakage during grinding (Lahtinen et al. 2014). Also, PFI mill refining was used as the pretreatment step and masuko grinder was used as a step to produce fine nanocellulose suspension (Trovagunta et al. 2021).

Hence in this work, an attempt has been made to produce nanocellulose using a disc refiner with a customised recirculation feedback arrangement for better process control and reduced energy consumption. The use of the milder a disc refiner is suitable for large scale nanocellulose film (nanopaper) formation, as it is much more energy efficient due to the geometry and high productivity (throughput) when compared with lab scale or grinding equipment.

The understanding of nanocellulose fiber diameter and its correlation with sheet strength has been well established. However the relationship between pore size distribution and barrier property as a function of energy consumption has not been established (Ang et al. 2019b). Refining reduces fiber size, and increases sheet density, and strength and reduces porosity. While numerous studies have been conducted which have focused on tailoring the structure and porosity of nanocellulose films by adding polymers and clays, there have been no studies reported which have investigated the trade-off between refining type, scale, level, and nanocellulose sheet barrier and optical property development. Nanocellulose is relatively cheap and can be obtained from biodegradable feedstocks like wood and other lignocellulosics rendering it highly suitable for sustainable barrier material development. There has been extensive work done based on the chemical composition of original starting pulp, mainly the hemicellulose content, which makes a difference in porosity, strength and barrier properties of the final nanopaper product (Chen et al. 2020; Kontturi et al. 2021; Taylor et al. 2020). However, there have been no investigations reported for nanocellulose which talks about scalability, energy consumption requirement for reaching good barrier properties comparable with plastic-based films. This research critically investigates the use of mechanical refining (PFI mill and disc refining) at various scales for nanopaper production and correlates the energy consumption with barrier and strength properties for the first time.

The embodied energy due to high electricity consumption during mechanical refining for producing nanocellulose has been reported to be around 108 MJ/kg (Nadeem et al. 2020). Other pre-treatments which can lower the energy consumption of producing nanocellulose are enzymatic, TEMPO oxidation and carboxymethylation, however there are challenges of toxicity and scale up via these methods (Isogai et al. 2011). The embodied energy of polymers like PP, HDPE, LDPE, PET (around 60–70 MJ/kg) is comparatively less than nanocellulose (Moon et al. 2013; Nadeem et al. 2020). Thus, for nanocellulose to replace existing polymers as packaging materials refining energy has to be decreased. Though addition of carboxymethyl cellulose (CMC) and montmorillonite (MMT) with nanocellulose has been reported to improve mechanical and barrier properties there has not been any study reporting the choice of pilot scale refiner to bring down the scale of energy consumption for nanocellulose production (Garusinghe et al. 2018; Nadeem et al. 2020).

Thus, in this work lab and pilot scale mechanical refining is conducted and evaluated to demonstrate nanopaper film property (mechanical, barrier) development in terms of packaging applications and energy efficiency of both the process is quantified.

### Materials and methods

The raw pulp used in our study were Bleached Eucalytptus pulp (BEK) that were supplied by Australian Maryvale with most of the lignin removed. The chemical composition of our BEK pulp was approximately 80% cellulose, 17% hemicellulose and 3% lignin (Ang et al. 2020).

#### PFI mill refining

A PFI mill (model no 164) was used to process hardwood BEK pulp, according to TAPPI standard 248 (TAPPI 2001a). Pulp at a solids content of approximately 10 wt% was refined at different refining levels and the refined samples were designated as BEK 15 k, BEK 30 k and BEK 50 k, respectively (Table 1). Unrefined BEK pulp was designated BEK 0. After PFI refining, the samples were diluted at 1.2 wt% and stored in a 2L jar. This is followed by disintegrating for 15,000 revolutions in a 3 L Mavis Engineering standard disintegrator (Model No 8522). A subsample equivalent of 1.2 gm dry weight is taken from the disintegrated sample storage jar which is diluted to 600 gm pulp suspension while passing through British hand sheet maker for 60 gsm sheet.

**Table 1:** BEK fiber sample name and refining level

Sample name	No of PFI milling revolutions
BEK0	0
BEK15k	15,000
BEK30k	30,000
BEK50k	50,000

#### Disc refining

A 14-inch disc refiner was used for processing which was powered by 112 kW motor. Pulp consistency of 3–4 wt% was maintained at a constant flow rate of 250–270 L/min. The plate gap geometry like including specific edge load (*SEL*) and bar edge length was held constant at 0.6 J/m and 2.74 km/rev, respectively. An automated Lab view software was used to control the process parameters (pressure, temperature, gap clearance, valve positions, flow rate) during refining trial. Unrefined NBSK fiber was labelled as NBSK0, and the most heavily refined pulp was named NBSK11 (Table 2).

**Table 2:** NBSK fiber sample name and refining level

Sample Name	Disc refining time, seconds
NBSK0	0
NBSK4	1260
NBSK6	1869
NBSK11	3959

#### Disc refining specific energy consumption

The specific energy consumption of Disc refiner was measured by Eq. 1 where net power ($Pnet$) is the total power used by the refiner motors minus the no-load power (water flowing through the refiner). The ratio of net power to the product of bar edge length *(*$BEL$*)* and refiner motor rotational frequency gives the specific edge load *(SEL)* (Eq. 2).

1 $$SEC = \frac{Pnet}{f}$$

where $Pnet$ is net power consumption in (kW), and $f$ is mass flow rate of dry fibers through refiners in (tonnes/h).

2 $$SEL = \frac{Pnet}{{BEL \times \left( {r/60} \right)}}$$

where $SEL$ is in J/m; $Pnet$ is in kW; $BEL$ (product of the total number of rotor and stator bars and contact length of opposite bars) is in km/rev; and $r$ is the refiner motor rotational frequency in revolutions per min. Table 2 summarises the name and refining levels for each sample.

#### Brecht-Holl fiber classification (screening)

Fines are characterised as subfraction of nanocellulose which passes through a 200-mesh screen. One of the lab-based screeners is Brecht-Holl Classifier fitted with a 200-mesh screen. Diluted pulp suspension (≤ 0.1 wt%) was used as a feed for dynamic Brecht Holl classifier, with a sufficient water jet velocity to prevent agglomeration of fibers. Fines passes through the screen while long fibers are retained on top of the mesh screen which are quantified gravimetrically. Fines were thus quantified by subtracting from long fiber mass fraction (Kargupta et al. 2021a). Approximately 1 gm dry weight of pulp sample was screened for each test.

#### Nanopaper making

The disintegrated samples at 0.2 wt% were used for nanopaper making at constant 60 gsm. The films were prepared by vacuum filtration (British handsheet maker, Model 8802) using a 22 µm pore size GE Whatman Grade 541 filter paper placed on top of 150 mesh screen. The drainage time during vacuum filtration was measured for each sheet. After draining, the sheet was couched manually by placing two new blotting papers and the couching plate (in the same order) on top of the filtered sheet and pressed according to TAPPI standard T205. This was followed by lifting the filter paper off the mesh, with the nanopaper, two blotting papers and the couching plate still on top of it. Then, it was placed on a table with the couching plate at bottom and the filter paper was carefully removed by hand from the nanopaper. The bottom blotting paper is replaced with a new blotting paper and a gloss pressing plate and another blotting paper was carefully placed on top of the nanopaper. This was followed by couching and pressing between two square metal plates at 345 kPa for 5 min in an automatic L&W sheet press (A B Lorentzen & Wettre, Model No 576).

Nanopaper making for each sample type was done in triplicate which was followed by room temperature drying at constant conditions (50% relative humidity, 23 °C according to TAPPI T402 standards (TAPPI 2001b) for 48 h prior to testing.

### Nanopaper property measurements

#### Mechanical properties

An Instron tensile equipment (Model No 5900) was used for stress strain measurements. Approximately 10–15 strips from 3 replicates were measured for each sample and refining type. The force (N) vs elongation at break (mm) was recorded for each strip. A strip with a width of 15 mm and test span of 10 cm was placed between two grips with a constant elongation rate (10 mm/min). Tensile index was plotted as a function of specific refining energy consumption. The stress vs strain curve was plotted to get the strain (%) at breaking load. The slope of the initial linear region of the curve gives the Young’s modulus. The strength results were expressed in terms of tensile index, Young’s modulus and strain at breaking load. A micrometer (Model No 51) was used to calculate the mean sheet thickness.

#### Barrier properties

The water vapour permeability (WVP) and water vapour transmission rate (WVTR) of the unrefined and refined NBSK and BEK films was measured at 23 ℃ and 50% relative humidity The nanopaper samples were pre-dried at 105 ℃ in a Thermoline BTC-9090 for at least 4 h prior to testing. 63.5 mm diameter cups complying with the standard were filled with dried calcium chloride (desiccant) and sealed with the test sample. The water vapour permeability is calculated based on rate of change of mass, which is then normalised by thickness of the paper to determine water vapour permeability (Maliha et al. 2020).

The oxygen permeability and oxygen transmission rate (OTR) through the refined nanocellulose sheet was determined using an Ox-Tran 2/22 oxygen transmission rate tester (Mocon) (ASTM 1927) at 23 °C and 50% relative humidity.

#### Fiber and nanopaper characterisation

The microscopic morphology of iridium coated air-dried fiber (0.001 wt%) samples was determined using Nova NanoSEM 450 FEG at an accelerating voltage of 5 kV. The unrefined fibers were imaged at a lower magnification of 200 ×–500 ×, and refined fibers were imaged at higher magnification of 50,000 ×. The image pixels were then calibrated with a length scale using Image J software, and the diameter of around 200 observable fibers were measured manually (Figs S2, S3) (Kargupta et al. 2021b).

The sheet porosity (%) and pore size distribution was measured using a Micromeritics AutoPore IV 9500 series porosimeter (Giesche 2006; Onur et al. 2018).

The sheet density was calculated by dividing the basis weight with mean thickness. The apparent porosity of the sheet was determined from Eq. (3) and cellulose density is assumed to be 1500 kg/m^3^ (Varanasi et al. 2013)

3 $${\text{Porosity}}\;(\% ) = \left( {1 - \frac{{{\text{sheet}}\,{\text{density}}}}{{{\text{cellulose}}\,{\text{density}}}}} \right) \times 100$$

An optical profilometer (OLS5000, Olympus, Japan) with a 50 × objective was used to determine the macroscopic morphology of nanopaper samples (259 µm × 259 µm area of both rough and smooth sides). The side of the nanopaper in contact with the stainless-steel side is the smooth side, and the side which was exposed to drying and blotting paper is referred to as the rough side. The smooth and rough side average surface aerial roughness (S_a_) and root mean square surface roughness (S_q_) were determined in triplicate (Figs. S5–S7).

An UV–vis spectrophotometer was used to study the nanopaper transparency in the visible light wavelength range.

### Results

#### Specific energy consumption (SEC)

The measured energy consumption was 0.37 kWh/tonne/rev for PFI refined BEK pulp and 0.846 kWh/t/s for disc refined NBSK pulp. For the most highly processed lab refined sample, BEK 50 k, this corresponded to an energy consumption of 17,417 kWh/t. NBSK 11, the most heavily disc refined sample, showed the highest specific energy consumption of 3346 kWh/t.

#### Effect of refining on fiber properties

**Fig. 1:**
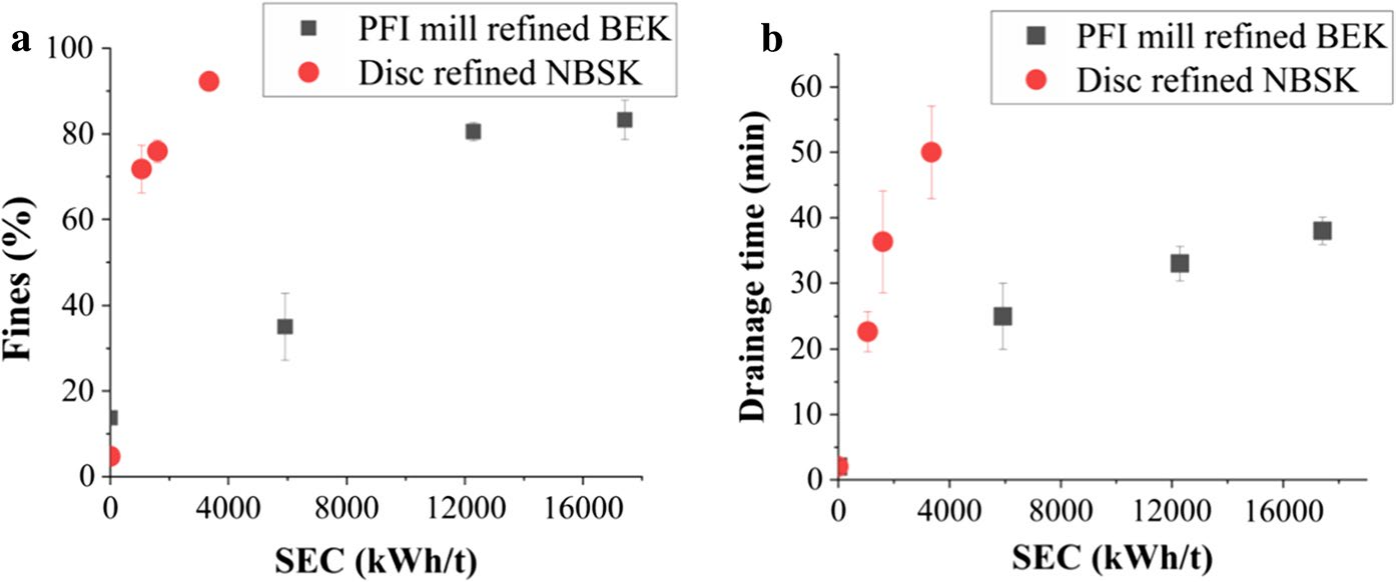
**a** Fines (%) and **b** Drainage time of nanocellulose as a function of energy consumption

Refining reduces fibre size with fines (Fibres passing through a 200-mesh screen) percentage reaching to 92.2 ± 1.0% and 83.2 ± 4.6% for NBSK 11 and BEK 50 k respectively (Fig. 1a). Given the mesh size of 76 µm, fines will be a mixture of nanofibres and larger fibres.

Generation of fines during refining also leads to increase in sheet drainage time since fines finer fibers reduces the size of the pores in the filter mat, which reduces the transport through them during sheet making (Fig. 1b). A 75–80% fines content in NBSK & BEK refined pulp comes at a cost of 12,292 kWh/t and 1600 kWh/t in PFI mill and Disc refining respectively. An increases of sheet drainage time is also evident at similar energy consumption.

**Fig. 2:**
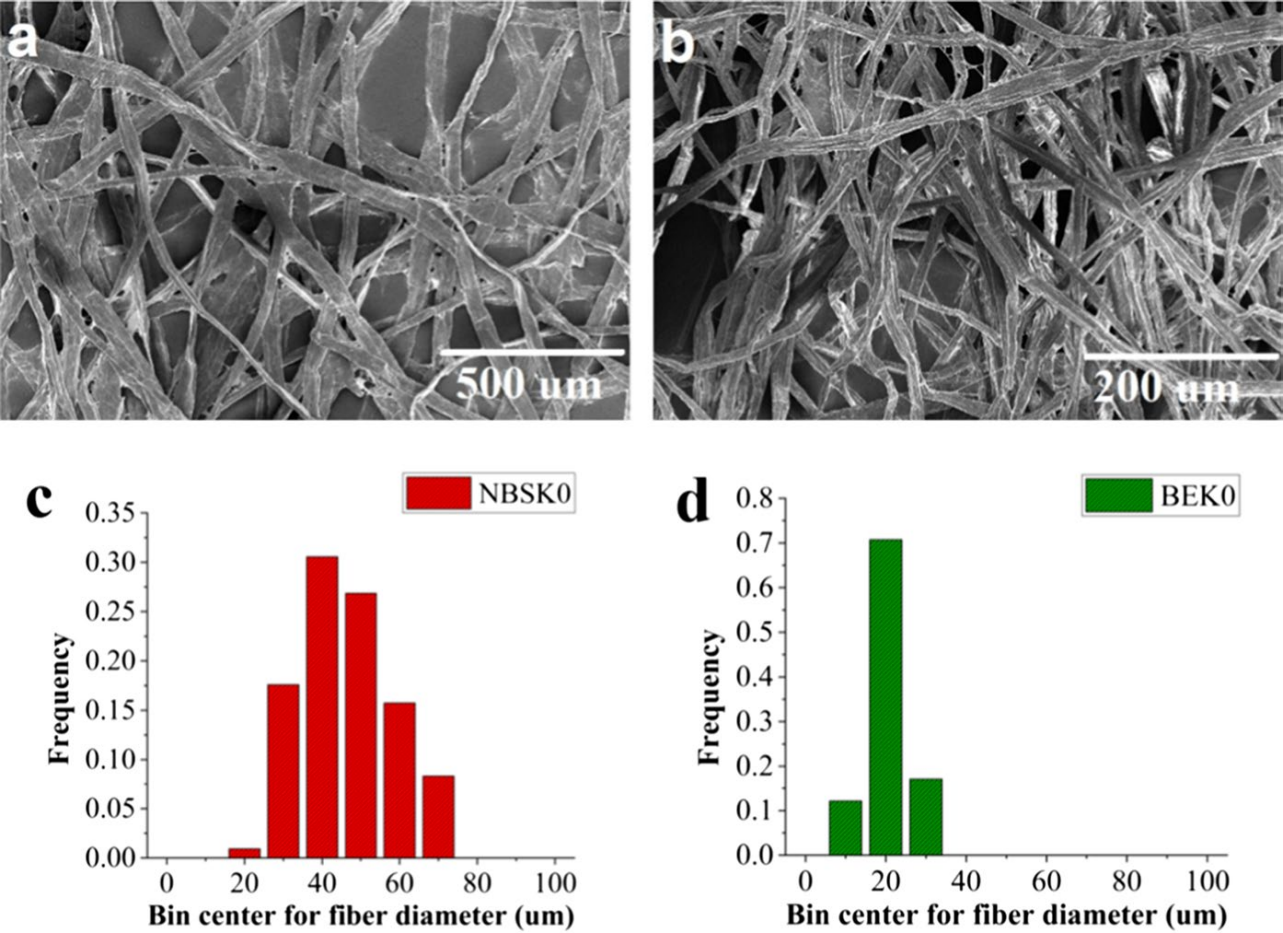
**a** Unrefined SEM images of **a** NBSK (NBSK 0) (Unrefined NBSK), **b** BEK (BEK 0), **c** NBSK0 diameter distribution and d) BEK0 diameter distribution

**Fig. 3:**
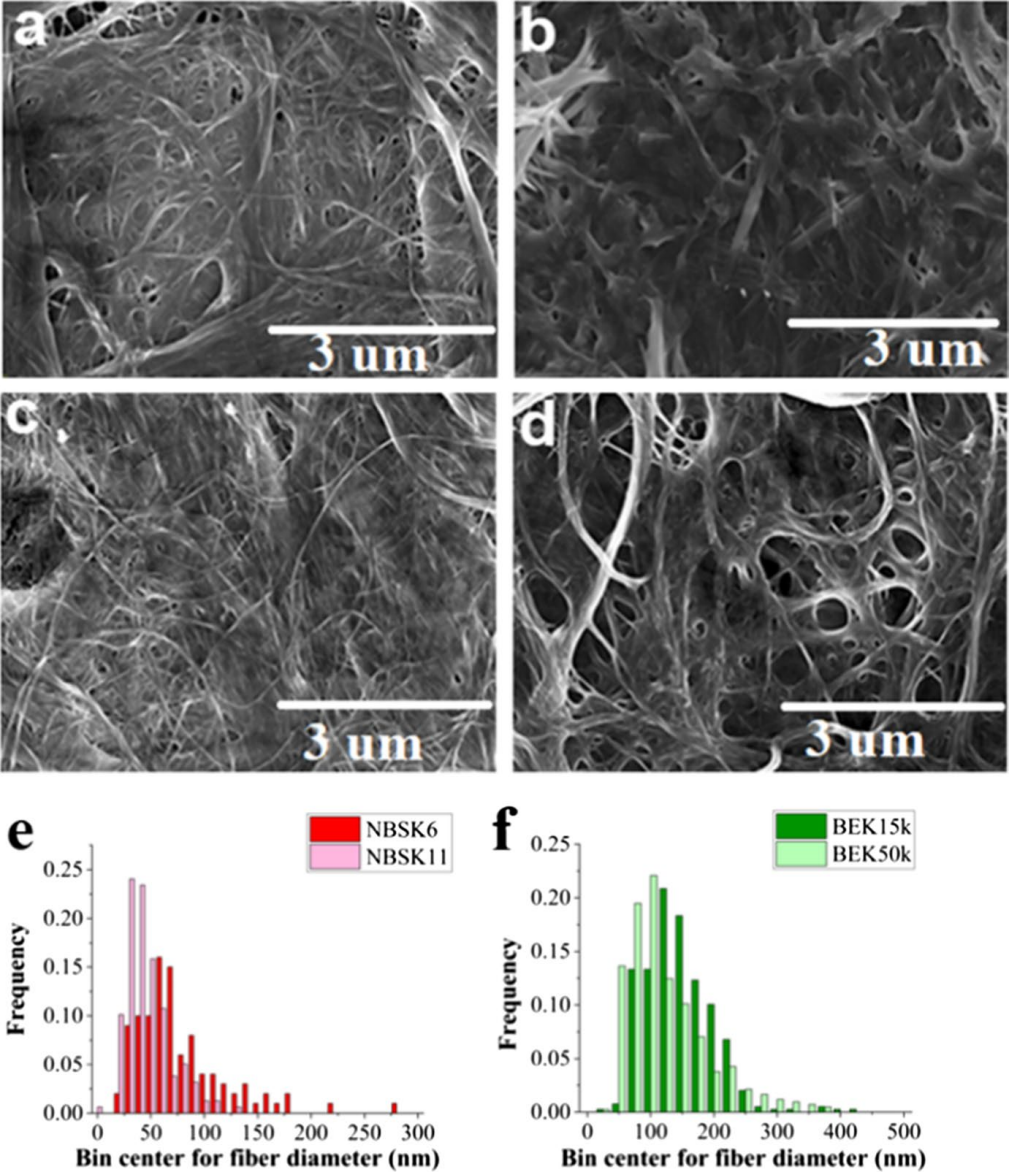
SEM images of **a** NBSK 6 (Medium refined NBSK), **b** BEK 15 k (Medium refined BEK), **c** NBSK 11 (Heavily refined NBSK) **d** BEK 50 k (Heavily refined BEK) **e** diameter histogram plots of unrefined NBSK6 vs NBSK11 and **f** diameter histogram plots of BEK 15 k vs BEK 50 k

Figure 2a, b depicts the SEM images of unrefined BEK and unrefined NBSK samples and Fig. 2c, d represents their fiber diameter histogram. Figure 3a and c shows the SEM images of medium and heavily disc refined NBSK, respectively, while Fig. 3b and d represent that of medium and heavily lab refined BEK fibers, respectively. The diameter distribution of medium and heavily disc refined NBSK and PFI mill refined BEK is compared in Fig. 3e and f, respectively. The combination of the SEC analysis with the fiber size analysis (Fig. S1 and Fig. 2), shows that disc refining required 3346 kWh/t (Fig. S1b) to reduce the median diameter of NBSK fibers from 40 µm (NBSK0) to 40 nm (NBSK11) (Figs. 2c and 3e). The input of 17,417 kWh/t SEC (Fig. S1a) in PFI mill refining reduces BEK median fiber diameter from 20 µm (unrefined) to 20 nm (BEK 50 k) (Figs. 2d and 3f).

Fiber diameter reduction from micron (Fig. 2a, b) to nanoscale enables the formation of a more compact and dense fiber network during sheet formation, which greatly reduces the accessible porosity (Fig. 3a–d). This clogging of pores through reduction of large fiber fraction (Figs. 2a, b and 3a, d) decreases the porosity of the fiber network, and enhances the strength of the nanocellulose sheet in comparison to a sheet formed with unrefined pulp fibers.

#### Effect of refining on nanopaper properties

In evaluating the potential of potential of nanocellulose in high-performance packaging applications we consider the relevant properties divided into barrier, mechanical and optical properties.

### Barrier properties

#### Mercury intrusion porosimetry (MIP) pore size distribution and porosity

**Fig. 4:**
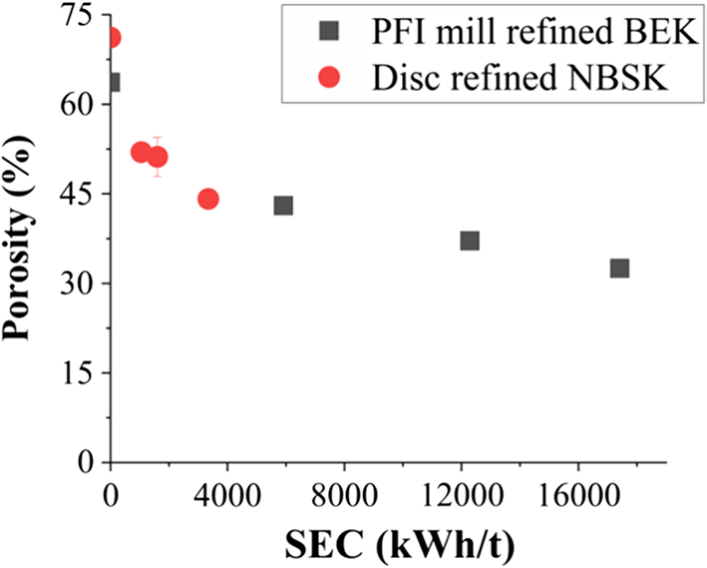
Porosity vs specific energy consumption

The effect of refining on porosity (%) and pore size distribution of nanopaper samples was studied by using mercury porosimetry. PFI mill refining for BEK and disc refining for NBSK significantly reduced the pore size distribution as (Fig. S4). Most heavily processed BEK and NBSK film reduced porosity approximately by 50% and 60%, respectively as compared with unrefined film (Fig. 4). Refining causes an increase in the number of fines (with smaller specific surface area) which act as fillers in the sheet, occupying the space of the pores, precisely where it is needed to significantly reduce the porosity of the porous unrefined film (Fig. 1a). Decreased porosity is strongly associated with improved barrier properties and mechanical strength of the nanopaper. The major driver of transport of gas and water vapor is the number and volume of pores in the nanopaper due to the high aspect ratio and higher surface area of the nanofibres. This kind of network decreases the permeability by increasing the nanocellulose sheet density. Thus, the extent to which diffusion through the pores within the sheet (tortuosity) can account for reduction in permeability by mechanical refining is a topic which deserves further research, and highlights the novelty of this work.

#### Water vapor and oxygen permeability

**Fig. 5:**
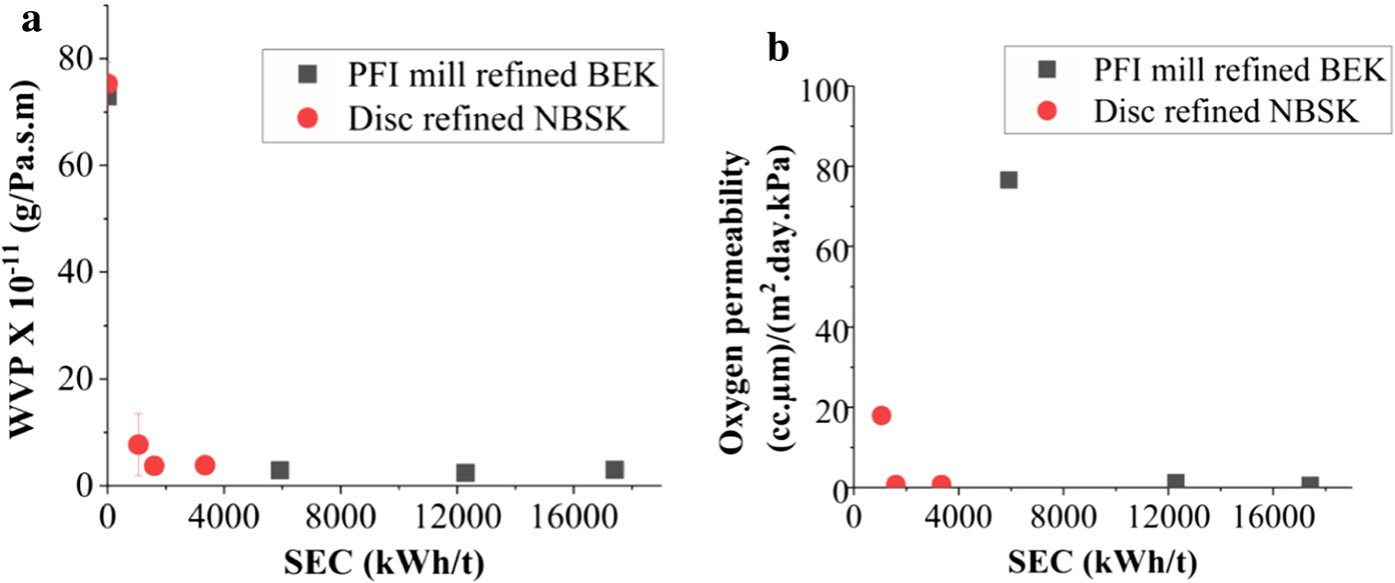
Water vapor permeability and oxygen permeability properties of nanocellulose films **a** WVP vs SEC **b** OP vs SEC

The water vapour was plotted as a function of refining energy consumption (Fig. 5a). There is approximately an order of magnitude reduction in water vapour permeability for refined as compared with unrefined film for both NBSK and BEK films. However, the rate of decrease reaches a threshold and there is not much improvement after certain level of refining. There is only a certain amount of refining energy (5917 kWh/t in lab PFI mill, 1600 kWh/t in pilot disc refiner) required to reach WVP of 3 × 10^–11^ g/Pa.s.m for both BEK and NBSK films (Fig. 5b).

Oxygen permeability was not detectable for unrefined films. It was measured first at 5917 kWh/t and at 512 kWh/t for PFI mill BEK and Disc refined NBSK respectively. Subsequently, the oxygen permeability of refined nanocellulose sheet decreased by 99.30% and 95.75% for BEK and NBSK respectively (Fig. 5b).

The oxygen permeability of refined nanocellulose sheet was plotted as a function of specific energy consumption and declining trend was observed to reach plateau (Fig. 5b). A threshold of oxygen permeability of approximately 1 cc µm/m^2^ day kPa was reached after 1600 kWh/t and 12,292 kWh/t in Disc and PFI mill refining respectively highlighting the suitability of Disc refining for sustainability and economic viability.

### Mechanical properties

**Fig. 6:**
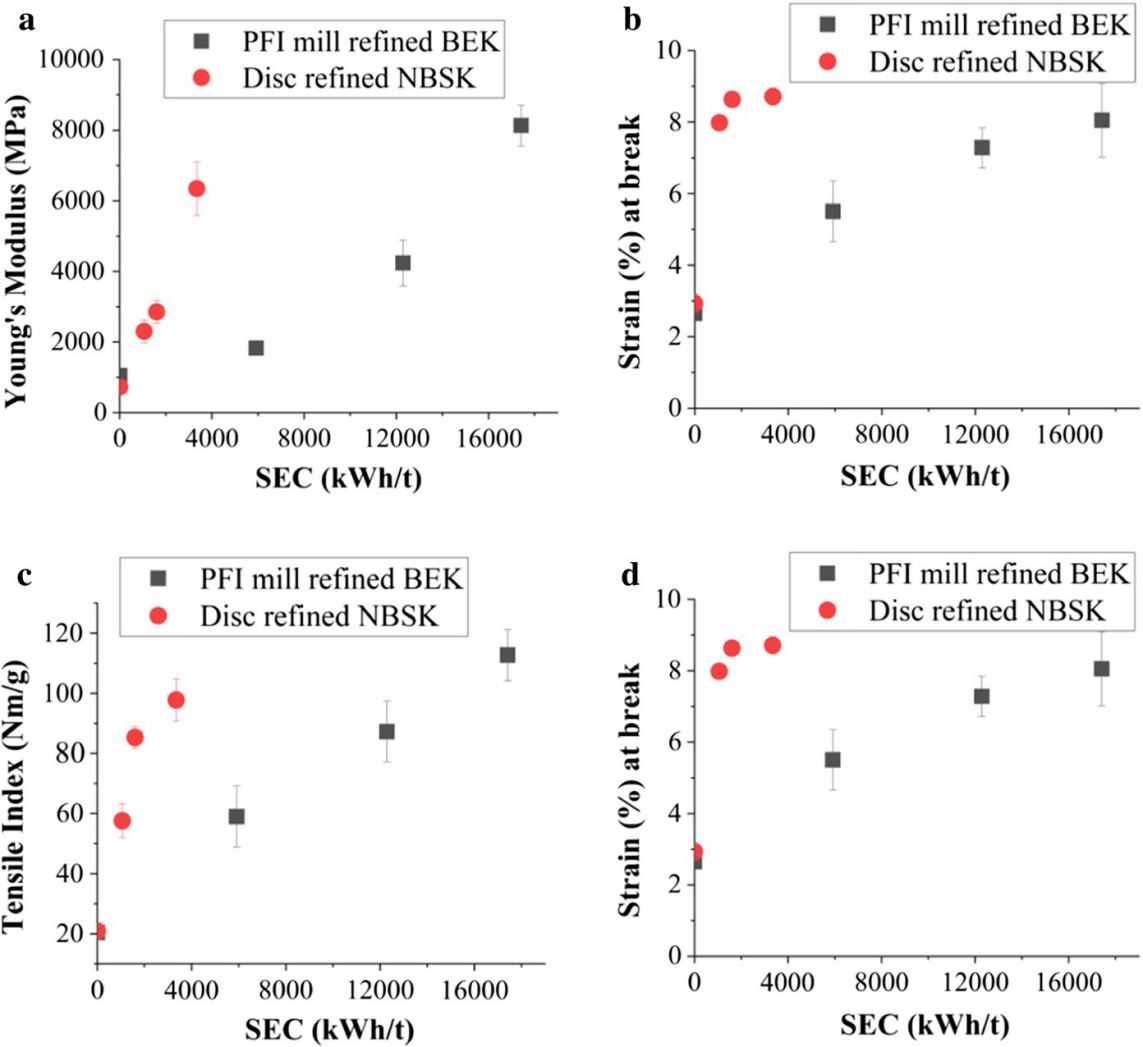
Mechanical property of nanocellulose films vs Specific energy consumption (PFI mill refined BEK, and Disc refined NBSK) **a** Young’s Modulus **b** Strain at break **c** Tensile Index **d** Sheet density

Figure 6a and b shows the elastic modulus and strain at break of nanopaper samples at various levels of refining. With an increase in specific energy consumption, there is an increase in both strain at breaking load and elastic modulus for both disc refined NBSK and PFI mill refined BEK. The breaking stress and sheet density data shown in the Fig. 4a, b has a similar trend. It takes approximately 17,417 kWh/t of specific refining energy to reach a Young’s modulus of 8134 MPa for PFI mill refined BEK. However, it only requires 3346.3 kWh/t of specific energy to reach 6344 MPa for Disc refined NBSK nanocellulose films. Tensile index achieved for the most heavily refined nanopaper was 112 Nm/g (PFI mill refined BEK) and 98 Nm/g (disc refined NBSK). This was approximately five-fold increase in strength when compared with unrefined fiber (Fig. 6c). With progressive refining the nanopaper density increases as the pores between fibers are filled by fines, which is also reflected in the approximately two-fold increase in sheet density (Fig. 6d).

### Optical properties

#### Surface roughness

**Fig. 7:**
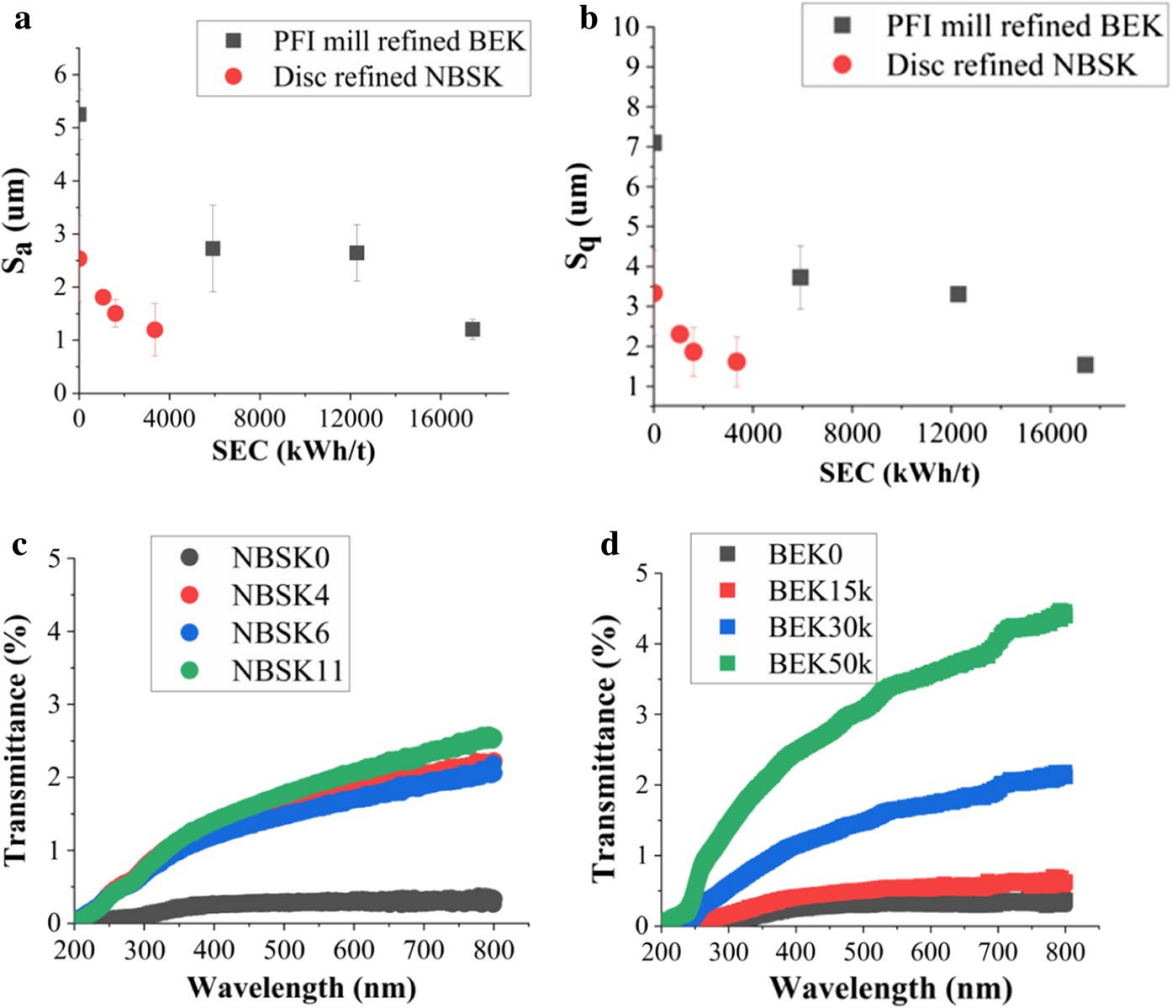
Smooth side Surface roughness as a function of SEC **a** Sa vs SEC **b** Sq vs SEC and **c** Transmittance of disc NBSK vs wavelength across various refining levels **d** Transmittance of PFI BEK vs wavelength across various refining levels

Another remarkable feature of refining is that it improves the smoothness of nanocellulose films. The surface roughness results from fiber coarseness and irregularities which result in vertical deviations from the true surface and can be quantified by aerial surface roughness (S_a_) and root mean square surface roughness (S_q_), with lower values indicating a smoother surface. These parameters were evaluated in triplicate from optical profilometer images, and the results are presented in Fig. 7. The optical profilometer images of the rough and smooth side of NBSK and BEK are also shown at various levels of refining in (Fig. S5). While S_a_ appears to be more statistically stable, S_q_ is more physically applicable as it provides information about peaks and valleys on the nanocellulose surface. Figure 7a and b show that to reach approximately 1 µm S_a_ and 1.5 µm S_q_ roughness, it requires 3346 kWh/t and 17,417 kWh/t of energy for disc refined NBSK and PFI mill refined BEK, resulting in a 77.05% and 52.8% decrease in surface roughness, respectively. The rough side roughness (S_a_, S_q_) was also measured, and shows similar trends (Figs S5–S7).

#### Optical transmittance

As fines percentage increases with increasing refining level, there is approximately 572% and 1136% enhancement in transmittance in the visible region (400 nm–700 nm), as compared with unrefined films for NBSK11 & BEK 50 k, respectively (Fig. 7c, d). This effect is due to the reduction in fiber diameter by refining from micro- to the nano-size range, which is below the wavelength of visible light (400–700 nm). Unrefined fiber dimensions are of micron scale and therefore scatter some or all of the visible light that is incident on them, as indicated in Fig. 7c and d.

## Discussion

The effect of refining on nanocellulose fibers and films were tested at a range of refining conditions for both disc refined NBSK and lab refined BEK. The nanocellulose (BEK and NBSK) sheet mechanical, barrier and optical properties were quite similar and comparable, however the energy consumption for Disc refining was almost five times less than the lab scale PFI mill refining.

Refining exposes the surface functional groups which increases hydrogen bonding and creates a more tortuous pathway for penetrating molecules, such as oxygen or water, significantly improving the sheet barrier properties. In this study, a decrease in median nanocellulose fiber diameter from 135 to 71 nm for BEK and 78–45.2 nm for NBSK (Fig. 3e, f) was observed with increased refining. The 3 orders of magnitude reduction in fiber diameter that occurs when cellulose fibers are broken down into nanofibers allows for production of nanometer sized cellulose fibers having excellent barrier properties. Most of the review papers on nanopaper have discussed the influence of range of structural, material and system process parameters on final property development (Barhoum et al. 2017; Benítez and Walther 2017; Xu et al. 2021). However, these investigators failed to quantify the relative energy consumption required to achieve different properties, which is critical for scaling up nanocellulose production. With increased refining tensile index and sheet density increased to above 100 Nm/g and 1000 kg/m^3^, respectively. A similar trend to this was observed with increasing grammage of nanopaper samples (Kontturi et al. 2021). Increases in tensile index and ductility have been also reported with an increase in hemicellulose content (Chen et al. 2020; Taylor et al. 2020) but for our samples the chemical composition was not varied. However, during mechanical refining there is an increase in mass fraction of fines which strengthens fiber–fiber bonding, explaining enhancement in sheet mechanical strength.

More mechanical energy consumption in refining leads to enhancement in both oxygen permeability and water vapor permeability, however there is a plateau beyond which there is no further improvement (Fig. 5). The plateau is achieved at relatively low energy inputs. For example, 5917 kwh/t and 1600 kWh/t are sufficient to achieve the WVP threshold of 3 × 10^–11^ g/m.s Pa for PFI mill refined BEK and disc refined NBSK, respectively (Table 4, Fig. 5a). Similarly, for oxygen permeability, 12,292 kWh/t and 1600 kWh/t are sufficient to achieve the OP threshold of 1 cc um/m^2^.day.kPa for PFI mill refined BEK and disc refined NBSK, respectively (Table 3, Fig. 5b). The diffusion process of the molecules through nanopaper samples happens in three steps: adsorption of the molecule onto the surface, followed by intermolecular diffusion through the sheet, and finally desorption from the opposite membrane surface (Nair et al. 2014). The rate limiting step is diffusion of the molecule through the fiber network. Refining cellulose fibers to nanocellulose decreases the porosity of the resultant films due to the substantial densification of the fiber network and decreasing the average pore size in the films. Thus, a dense low porosity nanocellulose sheet presents a more tortuous diffusion pathway for the diffusion of gas molecules than an unrefined cellulose fiber sheet. This is critical as network of pores in nanocellulose controls barrier performance. The extent to which tortuosity can account for reductions in permeability through refining is a topic that merits further research attention.

**Table 3:** Literature comparison of oxygen permeability of common materials used as coatings in packaging applications

Material	Oxygen permeability (cc µm)/(m^2^ day kPa)	Temperature & relative humidity (%)	References
Nanocellulose film	0.6	65%, RH 23 ℃	Österberg et al. (2013)
Nanocellulose (carboxymethylated)	0.85	50% RH, 23 ℃	Aulin et al. (2010)
Polyethylene (PE)	500–2000	50% RH, 23 ℃	Lange and Wyser (2003)
Polyethylene terephthalate (PET)	10–50	50% RH, 23 ℃	Lange and Wyser (2003)
Ethylene vinyl alcohol (EVOH)	0.01–0.1	50% RH, 23 ℃	Lange and Wyser (2003)
Low density polyethylene (LDPE)	1900	50% RH, 23 ℃	Aulin et al. (2010)
Experimental BEK & NBSK Nanocellulose sheet			
BEK15k	76.55	50% RH, 23 ℃	Present study
**BEK30k**	**1.244**	50% RH, 23 ℃	
BEK50k	0.533	50% RH, 23 ℃	
NBSK4	17.944	50% RH, 23 ℃	
**NBSK6**	**0.763**	50% RH, 23 ℃	
NBSK11	0.762	50% RH, 23 ℃	

The bolded values indicate the level of refining where the barrier properties reach a plateau

The potential impact of refining on the use of nanocellulose film as packaging material can be evaluated by comparing the barrier properties of the nanocellulose films with synthetic and other cellulose-based polymers. This is done in Table 3 for oxygen permeability and Table 4 for water vapour permeability. Also included in this table is some selected data from the literature for cellulose-based films. Table 3 shows that the plateau oxygen permeability of 1.24 cc µm/m^2^ day kPa is one to three orders of magnitude lower than the common petroleum-derived polymers, except for EVOH. However, the oxygen barrier properties of nanopaper samples are often compromised under high humidity conditions which may explain the observed order of magnitude difference in the oxygen permeability results (Tayeb et al. 2020). This contrasts with the water vapour permeability data in Table 4 where the plateau water vapour permeability of 3 × 10^–11^ g/m.s Pa is two to three orders of magnitude higher. Lowering this water vapour permeability further is one of the key challenges in improving the competitiveness of nanocellulose based packaging, especially since further refining or mechanical treatment would produce little further improvement. Possible approaches include producing composite films of refined nanocellulose with carboxymethyl cellulose (CMC) or montmorillonite (MMT), both of which have been shown to further enhance the barrier properties of nanocellulose films, bringing them closer to synthetic polymer performance (Garusinghe et al. 2018; Nadeem et al. 2020). This plateau in WVP occurs at relatively low energy levels which highlights the role and significance of pilot scale refining for energy efficient nanocellulose sheet production.

**Table 4:** Literature comparison of water vapour permeability of common materials used as coatings in packaging applications

Material	Water vapour transmission rate (g/m^2^ day)	Average film thickness (µm)	Water Vapour Permeability (g/m.s. Pa)	Testing conditions & References
Nanocellulose	234	42	8.12 × 10^–11^	Rodionova et al. (2011), 50% RH
Acetylated Nanocellulose	167	46	6.35 × 10^–11^	Rodionova et al. (2011), 50% RH
Polyethylene (PE)	16.8	18.3	1.00 × 10^–12^	Steven & Hotchkiss, (2002), 100% RH
Polyethylene terephthalate (PET)	16–23	25	7.78 × 10^–13^–1.12 × 10^–12^	Bhunia et al. (2013), 38 °C, 90% RH
Ethylene vinyl alcohol (EVOH)	22–124	25	1.07 × 10^–12^–6.032 × 10^–12^	Bhunia et al. (2013), 38 °C, 90% RH
Low density polyethylene (LDPE)	18	25	8.75 × 10^–13^	38 °C, 90% RH*
Experimental BEK & NBSK Nanocellulose sheet				
BEK0k	789.410	112	72.93 × 10^–11^	Present study, 50% RH, 23 °C
**BEK15k**	47.314	72	**2.91** × **10**^**–11**^	
BEK30k	44.783	65	2.39 × 10^–11^	
BEK50k	55.410	66	3 × 10^–11^	
NBSK0	571.816	160	75.3 × 10^–11^	
NBSK4	58.826	88	7.7 × 10^–11^	
**NBSK6**	50.350	86	**3.75** × **10**^**–11**^	
NBSK11	54.651	75	3.86 × 10^–11^	

The bolded values indicate the level of refining where the barrier properties reach a plateau

*Data taken from DuPont Teijin Films, http://usa.dupontteijinfilms.com

Figure 6 shows that refining improves mechanical sheet properties such as tensile index, sheet density, stiffness (Young’s modulus), and strain at break. Tensile Index and Sheet density increase with increased refining. Young’s modulus also increases continuously with refining, reaching 8 GPa for the PFI mill refined BEK. Strain at break also increased from less than 3% for both unrefined samples to over 8%.

This is mainly because during PFI mill and disc refining there are both external and internal fibrillation caused by compression effects. Internal fibrillation (peeling of primary and secondary cell wall) causes breakage of inner bonds making the fiber more flexible and increasing the bonding ability, which is a key factor in enhancing sheet density and tensile strength. External fibrillation generates nanofibres and fines, increases fibril area and the density of the network structure in the nanocellulose sheet, decreasing pore size and porosity (Fig. S4a, b, Fig. 4). Drainage time also increases for increasing refining level (Fig. 1b) due to smaller pore size created by the smaller fibers and nanofibers and consequently tighter fiber network formed during vacuum filtration. However, increased drainage time may have a detrimental effect on the papermaking process.

It should be noted that while Young’s modulus is comparable to that of PET and significantly higher than LDPE, the strain at break, even for the most refined sample, is at least an order of magnitude lower than petroleum-derived plastics where strains at break of 100–1000% are common. The optical properties are also significantly different to petroleum-derived plastics. While the nanocellulose film roughness decreases significantly with refining, the plateau value of ~ 1 µm is still significantly larger than, for example, LDPE, where surface roughness has been reported at ~ 20 nm (Čech Barabaszová et al. 2020). The much higher surface roughness of the nanocellulose film is also a significant contributor to the much lower visible light transmission of 0.9 for either LDPE or for PET (Wypych 2016; Yao et al. 2016). The transmission of nanocellulose films can be further improved by addition of additives, such as carboxymethyl cellulose (CMC) (Nadeem et al. 2020), although the transmittance will remain well below that of LDPE and PET.

Table 5 summarises the properties of the nanocellulose film made with this work and their position against the properties of LDPE and PET, selected as two common polymers used in packaging.

**Table 5:** Market potential of nanocellulose against two common exiting polymers (LDPE and PET)

Property	Unrefined BEK film (this study)	Nanopaper (this study)	LDPE	PET
Oxygen permeability	Above detection limit	Very low	Poor	Low
Water Vapour permeability	Very high	Low	Very low	Very low
Strain at break	Very low	Low	Very high	Very high
Elastic modulus	Low	High	Low	High
Transparency	Opaque	Partially translucent	High	Clear
Density	Low	Low-Moderate	Moderately High	Very High
Degradabality	Moderate	Low	High	High

Highly refined nanopaper can have replace the polyethylene layer in paper-polymer laminates, because of its reasonable barrier properties, resembling that of existing polymers, and because transparency is irrelevant for the polymer layer in such laminates. Also, it can be used as food packaging material to improve the shelf life of food while replacing existing non-biodegradable polymer. Most of the application of nanocellulose films have focused on composites which might have scalability problems, however in this research we demonstrate a simple, robust, scalable mechanical refining as an excellent tool to develop the nanocellulose sheet properties which can be used as a coating and filler which can be recycled and reused contributing to green economy. Our research demonstrates that refined nanocellulose can replace the existing polymers and plastics as packaging material where barrier and stiffness is critical, but transparency is not required (Table 5). Table 5 also shows that sheet density of refined nanocellulose remains moderately low to high which can be advantageous for high volume applications as packaging materials. Since barrier properties like oxygen and water vapor permeability are normalised by thickness, so an equivalent thick material prepared by nanocellulose will require less mass because it has low-moderate density as compared with PET. This can be advantageous for transportation of goods like packets of roasted nuts, washing powder, coffee beans, dehydrated foods, rice, and pasta where nanopaper can be a potential substitute for packaging. However, nanocellulose films prepared by refining alone will not be suitable for transparency and liquid packaging applications (Table 5).

Assuming most of the energy consumption of nanocellulose production happens during refining, a ten times energy efficiency is obtained through disc refining as compared with the production of conventional packaging films (Mohamed et al. 2014). Thus, considering energy consumption over packaging materials, nanocellulose produced at pilot scale could be more sustainable and it is certainly worthy in wide range of packaging applications. The embodied energy of disc refined NBSK will be substantially lower that of PET films.

Overall, if compared with PFI mill BEK pulp, disc refining consumes five times less energy in producing nanocellulose. The barrier properties of nanopaper samples reached a plateau at 5917 kWh/t in PFI mill and 1600 kWh/t in disc refining which highlights that high energy consumption is not necessary to produce nanocellulose with good barrier properties (Fig. 5). While production of nanocellulose has its own limitations with the required land use and significant water consumption for biomass production, it is crucial that the manufacturing production energy to final cost performance criteria for nanocellulose be properly addressed to compete with economies of synthetic packaging raw materials.

The high energy cost during mechanical refining is a serious threat for nanocellulose to become competitive with existing polymer. The embodied energy of virgin nanocellulose film produced by pilot scale refining obtained in this study was significantly lower than that of PET films (Nadeem et al. 2020). Producing nanocellulose in an energy efficient way have been tried out either by recycling or forming composites. However, disc refining has never been explored to bring down the embodied energy for nanocellulose production which again benchmarks the novelty of this work.

## Conclusion

Refining is a mechanical method of pulp fiber treatment that reduces cellulose to nanocellulose and improves the properties of nanocellulose film. The effect of mechanical refining is attributed to changes in the mechanical and geometrical properties of the fibers and fiber bonds due to internal fibrillation of the cell wall structure of cellulose. The increased conformability and flexibility due to refining leads to larger bonded areas, and stronger bonds between fibers, which enhances mechanical and barrier property. At 17,417 kWh/t SEC PFI mill refined BEK achieved a young’s modulus of approximately 8134 MPa however it took only 3346.3 kWh/t SEC to achieve a similar result for disc refined NBSK. Fiber size reduction (from micron to nanoscale), which increased sheet drainage time, also enhanced the barrier properties of the nanopaper samples both in terms of oxygen and water vapour permeability. More fibrillation decreased the mean fiber diameter of both NBSK and BEK pulp by three orders of magnitude, significantly decreasing sheet porosity and increasing the tortuosity of the diffusion path for a permeating molecule, which improves the barrier properties of refined nanocellulose films. The water vapour permeability is low for nanocellulose, but it is still two orders of magnitude higher than that of LDPE so not comparable. However, the oxygen permeability values achieved were comparable with those of synthetic polymers used in packaging applications, and thus the refining of cellulose represents a potentially sustainable, renewable replacement material for the packaging industry. There is also a plateau observed for barrier properties, which means a trade-off can be achieved between energy consumption and product application for the sustainable production of nanocellulose.

## Supplementary Information

Below is the link to the electronic supplementary material.

Supplementary file1 (DOCX 2216 kb)
