# Supplementary material for: Preparation and benchmarking of novel cellulose nanopaper
Source: Cellulose (Lond). 2022 Apr 15;29(8):4393–411. doi: 10.1007/s10570-022-04563-0 (PMC9012250; doi:10.1007/s10570-022-04563-0)
Supplement: Supplementary file 1 — Supplementary file1 (DOCX 2216 kb) [file 10570_2022_4563_MOESM1_ESM.docx]

Supplementary Information

for

**Preparation and benchmarking of novel cellulose nanopaper**

Wriju Kargupta ^a^, Reanna Seifert ^b^, Mark Martinez ^b^, James Olson ^b^, Joanne Tanner ^a^, Warren Batchelor ^a, *^


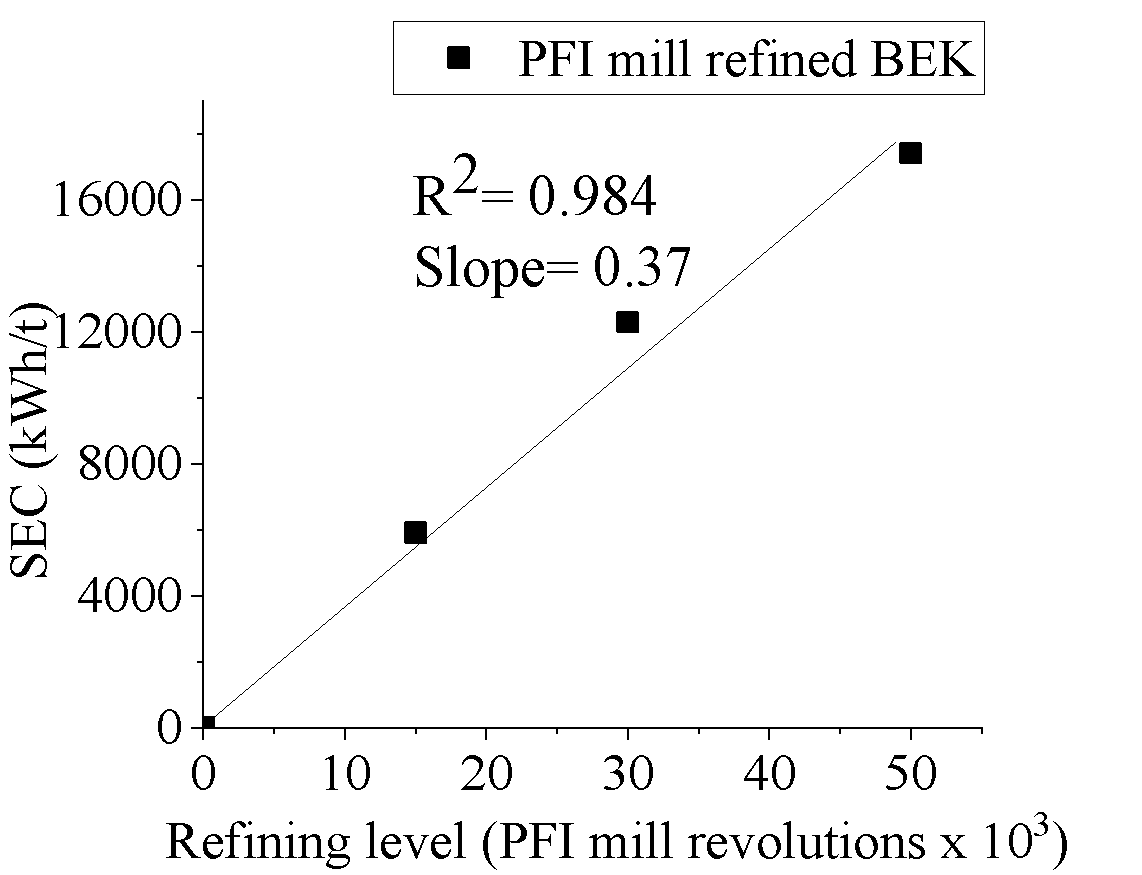


**Figure S1a: PFI mill BEK energy consumption versus refining level**


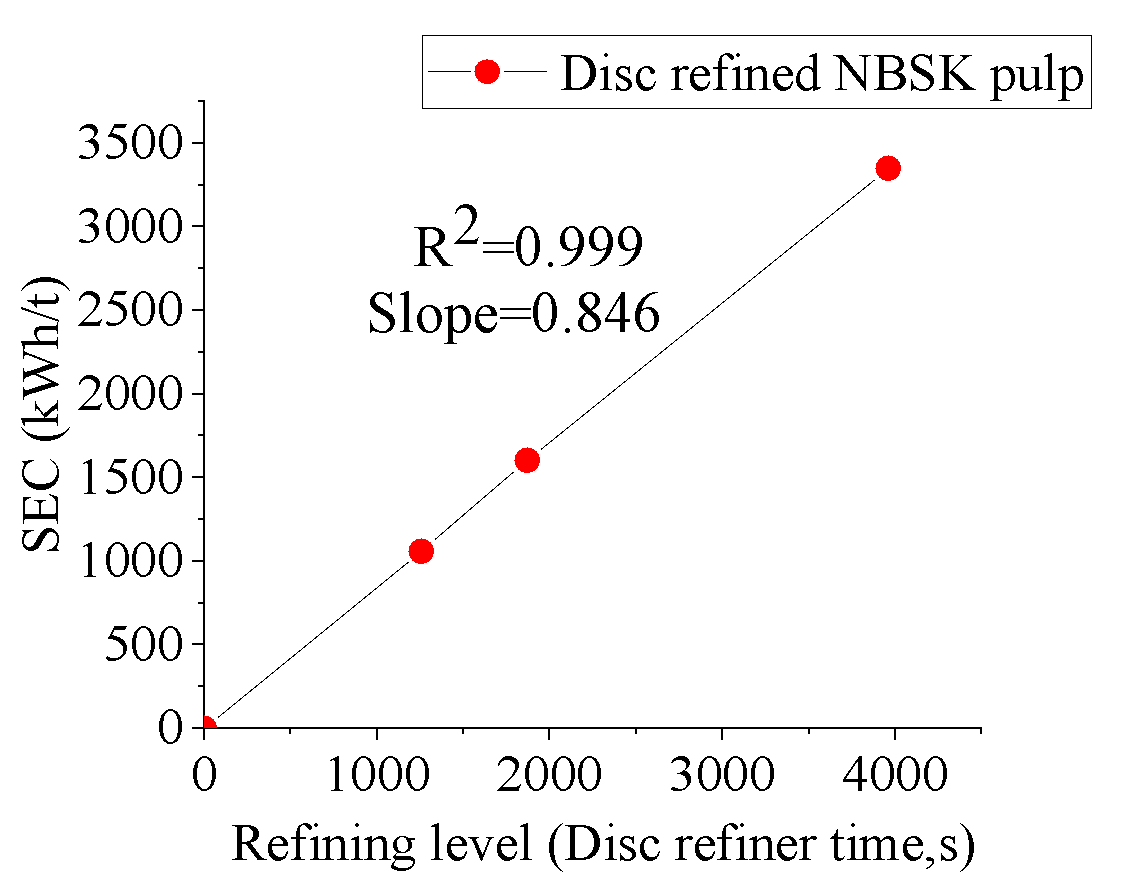


**Figure S1b: Disc refining NBSK energy consumption versus time**


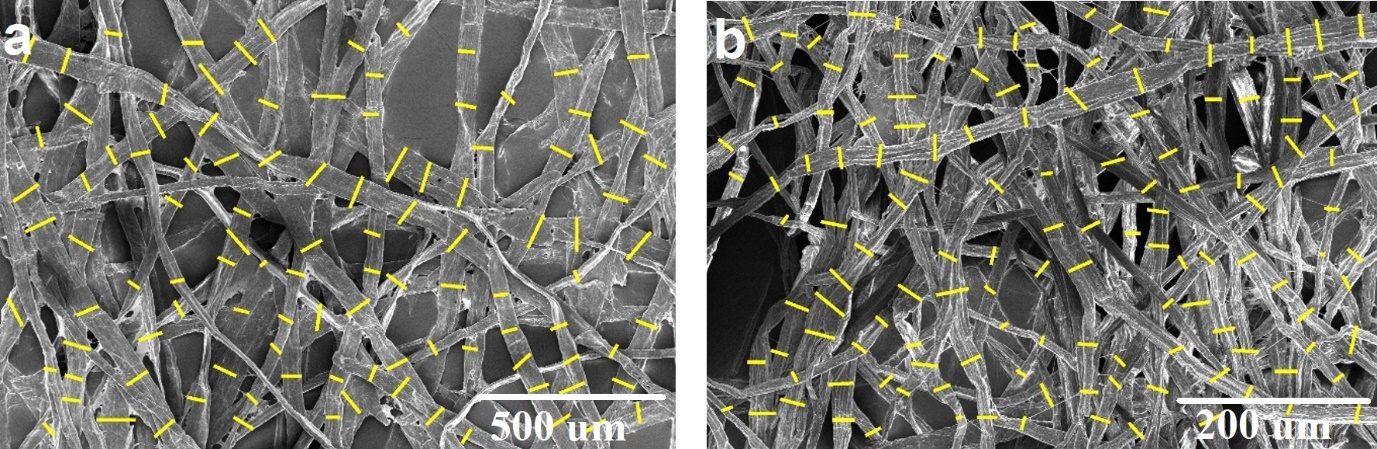


**Fig S2: Annotated Unrefined SEM images of a) NBSK0 and b) BEK0**


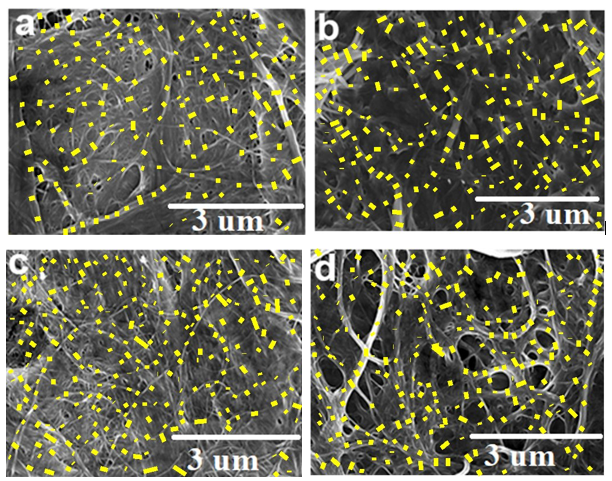


**Fig S3: Annotated SEM images of a) Medium disc refined NBSK (NBSK6) and b) Medium PFI mill refined BEK (BEK15k), c) Heavily disc refined NBSK (NBSK 11) and d) Heavily PFI mill refined BEK (BEK 50k)**


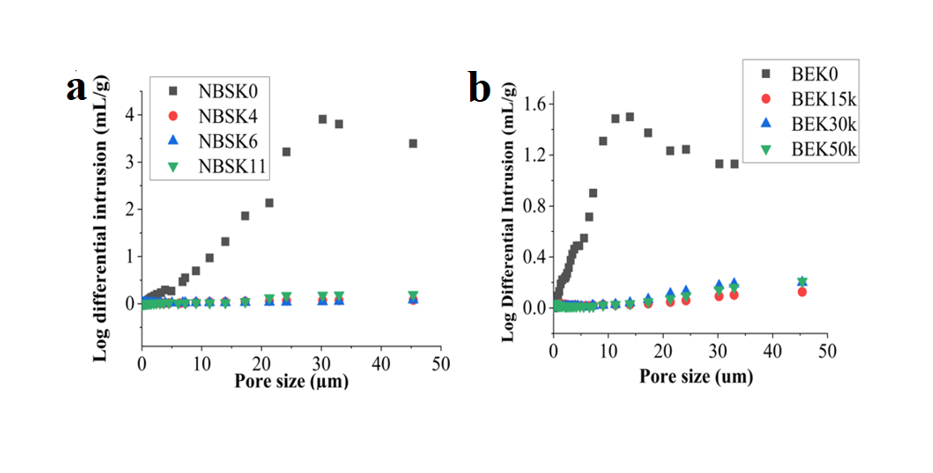


**Fig S4: Porosity vs SEC a) Disc NBSK and b) PFI BEK**


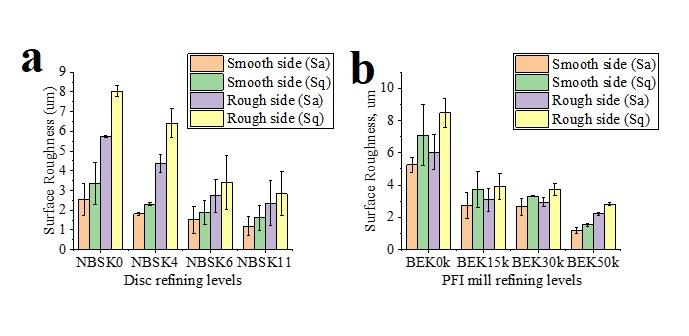


**Fig S5: Surface roughness vs Refining levels for a) Disc refining b) PFI mill refining**

| **PFI mill Refining Level** | **Smooth Side of NC film** | **Rough Side of NC film** |
| --- | --- | --- |
| BEK0 | 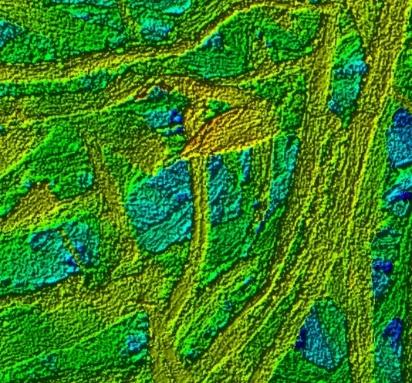 | 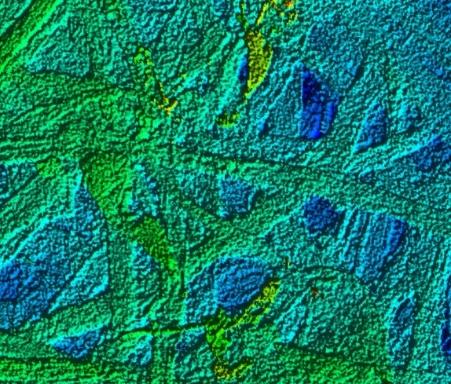 |
| BEK15k | 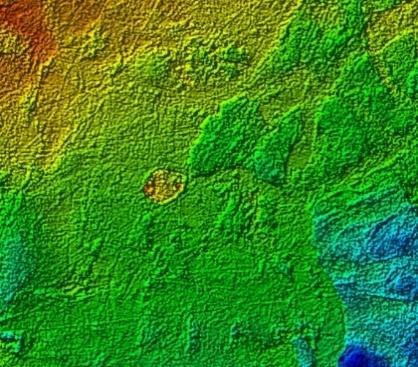 | 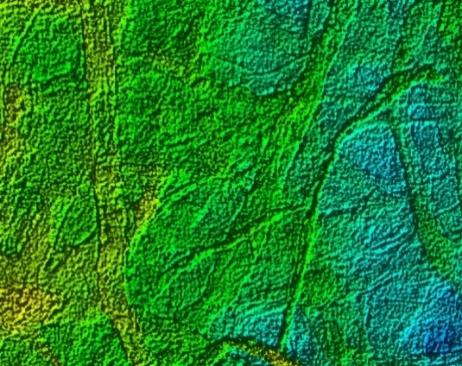 |
| BEK30k | 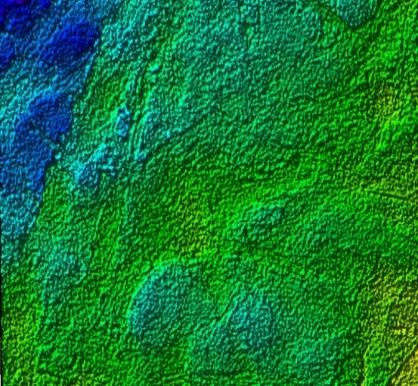 | 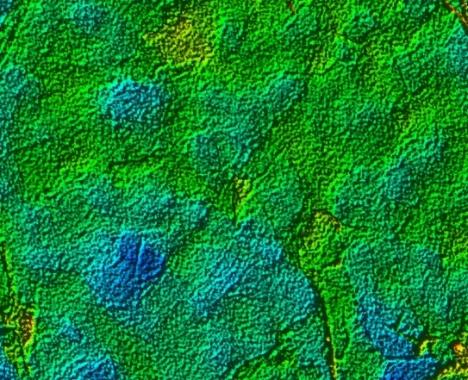 |
| BEK50k | 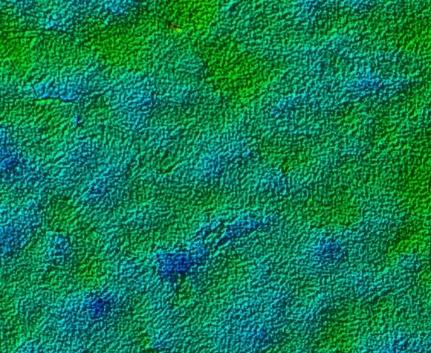 | 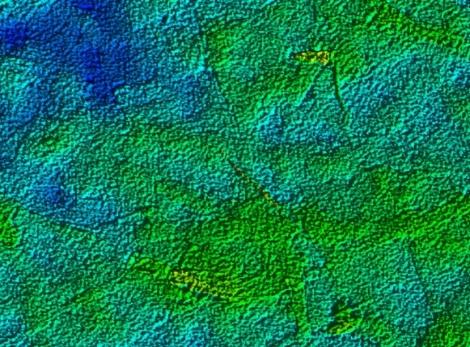 |

**Figure S6: Optical Profiler Images of NC sheets from Vaccum Filtration at various PFI mill refining level. 50x Magnification.**

| **Disc Refining Level** | **Smooth Side of NC film** | **Rough Side of NC film** |
| --- | --- | --- |
| NBSK0 | 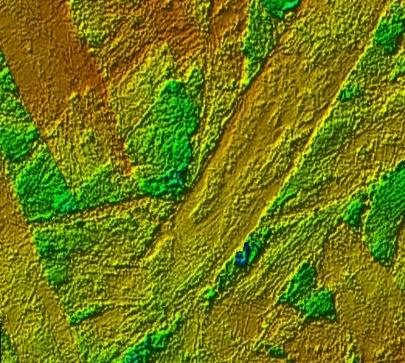 | 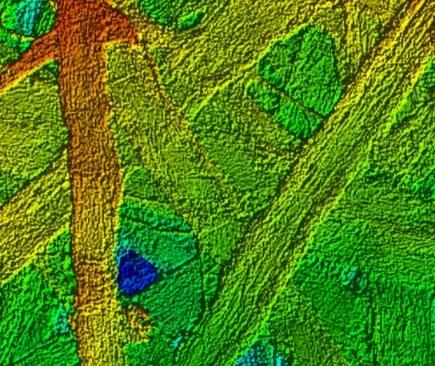 |
| NBSK4 | 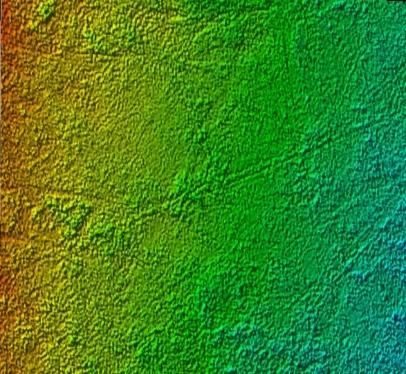 | 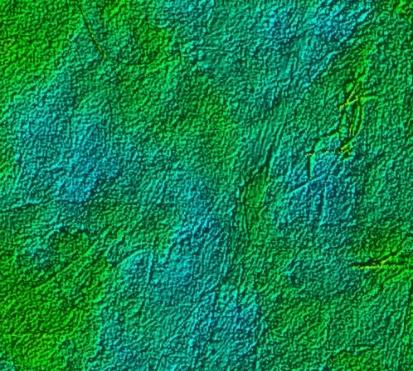 |
| NBSK6 | 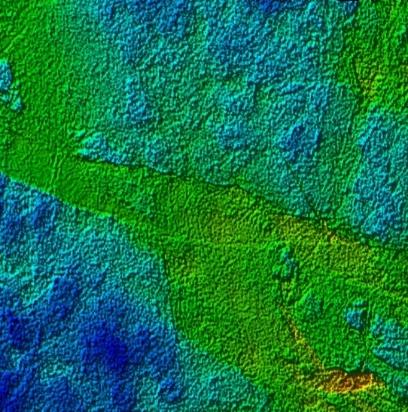 | 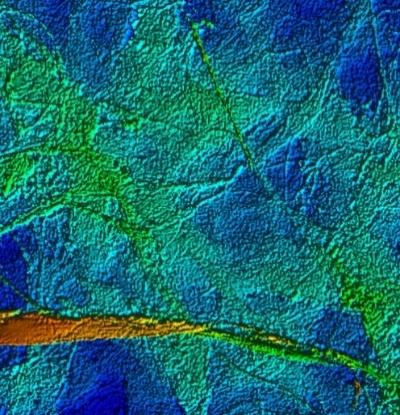 |
| NBSK11 | 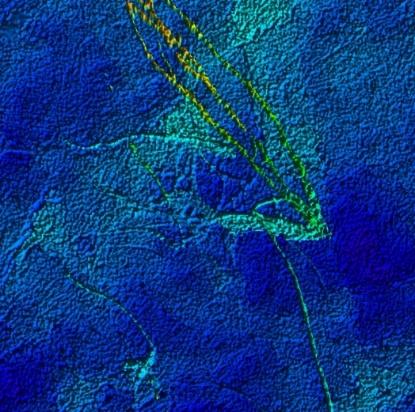 | 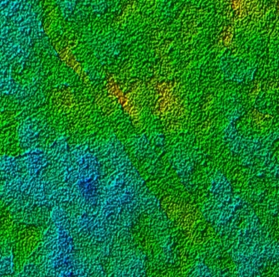 |

**Figure S7: Optical Profiler Images of NC sheets from Vacuum Filtration at various PFI mill refining level. 50x Magnification.**
